# Effect of Recombinant Spidroins Self-Assembly on Rheological Behavior of Their Dispersions and Structure of Electrospun Nanofibrous Materials

**DOI:** 10.3390/polym15143001

**Published:** 2023-07-10

**Authors:** Timur Kh. Tenchurin, Roman V. Sharikov, Sergei I. Belousov, Dmitry R. Streltsov, Sergey N. Malakhov, Evgeny V. Yastremsky, Yuri M. Chesnokov, Lyubov I. Davydova, Vladimir G. Bogush, Sergei N. Chvalun

**Affiliations:** 1 National Research Center “Kurchatov Institute”, 123182 Moscow, Russia; 2 Enikolopov Institute of Synthetic Polymeric Materials, Russian Academy of Sciences, 117393 Moscow, Russia; 3 Shubnikov Institute of Crystallography, Federal Research Center Crystallography and Photonics, Russian Academy of Sciences, 119333 Moscow, Russia

**Keywords:** biopolymers, recombinant spidroin, IR spectra, electrospinning, nonwovens

## Abstract

The effect of primary amino acid sequence in recombinant spidroins on their spatial organization is crucial for the fabrication of artificial fibers and fibrous materials. This study focuses on the rheological properties of aqueous and alcoholic solutions of recombinant analogs of natural spidroins (rS1/9 and rS2/12), as well as the structure of their films and nanofibrous materials. Non-Newtonian flow behavior of aqueous solutions of these proteins was observed at certain concentrations in contrast to their solutions in hexafluoroisopropanol. The secondary structure of recombinant spidroins was addressed by IR spectroscopy, whereas their self-organization in various solvents was studied by AFM and cryo-TEM. The influence of the solvent on the structure and properties of the films and nanofibrous materials produced by electrospinning has been established.

## 1. Introduction

Spidroin proteins, which comprise the skeletal filament of the orb-web spider web, are distinguished by their high molecular weight (>300 kDa) and a unique combination of high strength and elasticity [1]. The structure of spidroin molecules is represented by a central amphiphilic domain (4000–20,000 amino acid residues) and two non-repeating hydrophilic regions (100–150 amino acid residues) at the ends of molecules [2]. The central amphiphilic domain consists of repeating amino acid motifs enriched in glycine and contains poly-Ala blocks of 5–8 amino acid residues in length. The poly-Ala blocks are folded into β-sheets with the formation of extensive hydrogen bonds. During the formation of spider web threads, the poly-Ala blocks are able to assemble into crystalline regions that provide the strength of the spider silk. Glycine-rich regions form an amorphous matrix, which imparts elasticity to the spider web fibers. The amorphous matrix is represented by α-helices, non-oriented regions, and β-turns [3]. The hydrophilic terminal regions of spidroin molecules maintain the protein in a soluble state in the lumen of the spider gland and are involved in the correct formation of the filaments [4].

High biocompatibility and an advantageous combination of high strength and elasticity of the spidroin fibers make them attractive for medical applications and for the fabrication of composite materials [5]. Unfortunately, it is impossible to produce spider silk using the mass breeding of spiders. Therefore, in recent years, recombinant analogues of natural spidroins have been developed. They are synthesized primarily by recombinant DNA technology using plants, goats, silkworm caterpillars, yeast cells, and bacteria [6,7]. The materials based on recombinant spidroins (RS) are promising for use in pharmaceutical technology as drug carriers, in tissue engineering, and in soft optics and electronics [8]. The RS-based matrices are currently successfully used as cell scaffolds for the proliferation and differentiation of various types of cells [9,10]. The fibrous materials fabricated by electrospinning from the RS solutions have a structure and mechanical properties that mimic the natural extracellular matrix [11,12], which is of considerable interest for tissue engineering.

RS usually contain a large number of primary repeats characteristic of the central part of natural spidroins, but they have a lower molecular weight. Because the N- and C-terminal sequences are absent in most RS, they have very low solubility in water, i.e., from 0.4 up to 2% [12]. Therefore, for the dissolving RS, organic solvents are mainly used, e.g., hexafluoroisopropanol (HFIP). The disadvantages of HFIP include its toxicity, high cost, and the need for subsequent treatment of the fabricated fibrous materials with ethanol to improve their mechanical characteristics [3]. A promising method for dissolution of RS in water using microwave heating has been proposed by Jones et al. [13]. As a result, the authors reported almost complete dissolution of the protein with a high concentration (up to 12%) at a temperature of ~130 °C without its degradation. They also prepared hydrogels, films, sponges, and microfibers from RS. The solubility of proteins in water depends on their electric charge, the ratio of polar and non-polar groups on the surface of the proteins, and their molecular weight. Jones et al. used relatively low molecular weight spidroin (M ≤ 50 kDa), and they did not use electrospinning for the formation of a nonwoven material. It should be noted that the use of high molecular-weight proteins can help to improve the interchain interaction and reduce the number of defects in the structure of the material, which should enhance its mechanical properties. In this study, we focus on the structure of RS rS1/9 and rS2/12 spidroins (with a molecular weight of 94 and 113 kDa, respectively) in formic acid and HFIP solutions synthesized from cells of the yeast strain-producers *Saccharomices cerevisiae*, as well as on the structure of their films and fibrous materials. Understanding the effect of the structure of these proteins on the rheological characteristics of their spinning solutions is crucial for the fabrication of artificial fibers and fiber materials with desired properties using electrospinning.

## 2. Materials and Methods

### 2.1. Synthesis of Recombinant Spidroins

The RS genes rS1/9 and rS2/12 were synthesized earlier and cloned in yeast *S. cerevisiae* cells [14]. The rS1/9 protein is characterized by a molecular weight of 94 kDa and consists of nine so-called monomers. Each monomer contains four primary repeats and has the following sequence:

SQGAGQGGYGGLGSQGAGRGGLGQGAGAAAAAAGGAGQGGLGGQGAGQGAGAAAAAAGGAGQGGYGGLGSQGAGRGGLGQGAGAAAAAAAGGAGQGGYGGLGSQGAGRGGQGAGAAAAAAGGAGQGGYGGLG

The rS2/12 protein has a molecular weight of 113 kDa, contains 12 repeats of the monomer, which consists of 5 primary repeats, and has the following sequence:

GPGGYGPGQQGPGAAAAASAGRGPGGYGPGQQGPGGSGAAAAAAGSGPGGYGPGQQGPGGPGAAAAAAAGRGPGGYGPGQQGPGGSGAAAAAAGRGPGGYGPGQQGPGGPGAAAAAA

Production of the biomass of yeast producers *S. cerevisiae* SCR_702T-rS1/9 and SCR-702T-rS2/12, isolation from the water-insoluble cell fraction, and purification of RS rS1/9 and rS2/12 were performed as described previously [15], with some modifications. In particular, at the stage of extraction of the desired proteins from fragments of the yeast cells after destruction, a stage of preliminary extraction with glacial acetic acid (extraction of ballast proteins) was introduced, followed by extraction of RS with a solution of 10% lithium chloride in 90% acetic acid. Further procedures have been described previously [15]. After purification, the proteins were dialyzed against deionized water, frozen at −70 °C, and freeze-dried.

### 2.2. Atomic Force Microscopy

Dispersions of spidroins rS1/9 and rS2/12 (0.08 mg/mL) in HFIP (Sigma-Aldrich, St. Louis, MO, USA, ≥99%) and in 2% formic acid (Sigma-Aldrich, St. Louis, USA, ≥98%) aqueous solutions were prepared. A drop of the dispersions (20–30 μL) was applied to a freshly cleaved mica substrate and kept for 2 min. Afterward, the substrates were rinsed with the solvent used (i.e., HFIP or formic acid solution) for 5 min and dried overnight in the air at room temperature.

The AFM measurements were performed in the PeakForce Tapping QNM mode in air at room temperature on a Multimode 8 microscope (Veeco, Santa Barbara, CA, USA) with a Nanoscope V controller (Veeco, Santa Barbara, CA, USA). Silicon nitride cantilevers SNL10 (Bruker, Santa Barbara, CA, USA) with a nominal resonant frequency of 65 kHz and a force constant of 0.35 N/m were used as the AFM probes.

### 2.3. Rheological Studies

For rheological studies, dispersions of spidroins rS1/9 and rS2/12 (3, 11, and 50 mg/mL) were prepared in aqueous solutions of 2% and 5% formic acid. The solutions (3 and 80 mg/mL) of these proteins in HFIP were also prepared. The rheological properties of the dispersions and solutions were studied on a Physica MCR 501 rheometer (Anton Paar GmbH, Graz, Austria) in the shear and oscillatory deformation modes. As measuring geometries, a double-gap cylindrical system (DG 26.27/T200/SS) or a cone/plane system (CP25-1/TG, angle 1.070, diameter 25 mm) were used depending on the viscosity of the samples. The temperature was 20 °C. To prevent solvent evaporation, the sample surface was coated with a thin layer of low-viscosity mineral oil. Before carrying out the oscillation measurements, the region of linear viscoelasticity (LVE) was determined.

### 2.4. Film Casting

To study the effect of the solvent on the secondary structure of the proteins, the films of RS rS1/9 and rS2/12 were cast from dilute dispersions and solutions with a concentration of 1 to 10 mg/mL in a 5% formic acid solution and HFIP.

### 2.5. Electrospinning

The preparation of nanofibrous materials by electrospinning was carried out from dispersions of rS1/9 and rS2/12 in HFIP (80 mg/mL) and from a dispersion of rS2/12 in 5% formic acid solution (11 mg/mL or 19 mg/mL with poly(ethylene oxide) (PEO, Mw = 2000 kDa, Merck, Rahway, NJ, USA) as an additive. A voltage in the range of 18 to 26 kV was applied to the capillary, the volume flow rate was 0.8 mL/h, and the distance between the electrodes was 25 cm. The materials were collected on the grounded stationary electrode. After fabrication, some of the fibrous samples fabricated from the HFIP dispersions were placed in 96% ethanol for 30 min to study conformational changes in the proteins. After preparation, all the samples were placed in a vacuum cabinet for 1 day to remove the remaining solvent.

### 2.6. Scanning Electron Microscopy of the Fibers

The surface morphology of the RS fibers was studied using a FEI Versa 3D Dual Beam Scanning Electron Microscope (Thermo Fisher Scientific, Hillsboro, OR, USA) in high vacuum mode. The SEM images were recorded using a secondary electron detector at an ultra-low accelerating voltage of 1 kV. The analysis of fiber lengths and diameters, as well as their distributions, was carried out using the Fiji software package. The average fiber diameter was calculated from the measurements of at least 100 fibers.

### 2.7. Transmission Scanning Electron Microscopy

The structure of RS solutions in HFIP was addressed using the STEM-in-SEM technique on a FEI Versa 3D Dual Beam microscope (Thermo Fisher Scientific, Hillsboro, OR, USA) at an accelerating voltage of 30 kV and a beam current of 70 pA. The RS solutions with a concentration of 3 mg/mL were preliminarily contrasted with a 1% uranyl acetate solution and deposited on a carbon-coated TEM copper grid. The images were recorded using a circular STEM detector in bright field mode.

### 2.8. Cryoelectron Transmission Microscopy

The RS dispersions (3 mg/mL) in a 5% formic acid solution were distributed in an even layer over the surface of a carbon-coated TEM copper grid pretreated with a glow discharge in a PELCO easiGlow™ (Ted Pella, Northport, NY, USA) for 30 s at a plasma current of 25 mA. The applied sample dispersion (3 μL) was abruptly frozen in liquid ethane after being blotted with filter paper for 1.5 s using a Vitrobot Mark IV (Thermo Fisher Scientific, Hillsboro, OR, USA) to form amorphous ice. The images were acquired on a Titan Krios 60-300 (Thermo Fisher Scientific, Hillsboro, OR, USA) cryoelectron transmission microscope equipped with a spherical aberration corrector (CEOS, Heidelberg, Germany) at an accelerating voltage of 300 kV in low dose mode using EPU software (Thermo Fisher Scientific, Hillsboro, OR, USA).

### 2.9. IR Spectroscopy of the Films and Nonwovens Materials

The films and nonwoven materials were studied by IR spectroscopy using a Thermo Scientific Nicolet S5 IR Fourier spectrometer with an iD5 ATR attachment. Deconvolutional analysis of the amide I band (1700–1600 cm^−1^) was performed using the Origin 2016 software. The IR spectra were smoothed using the Savitsky–Golay method (9-point window and a second-degree interpolating polynomial). The detection of peaks was carried out by analyzing the characteristic points of the second derivative. The peak half-width was fixed in the range from 10 to 30 cm^−1^ [16].

## 3. Results

### 3.1. Rheological Properties of the RS Dispersions

The rheological behavior of the RS rS1/9 and rS2/12 dispersions in formic acid aqueous solutions and in HFIP was studied to obtain insight into their structure and conformational transitions during formation under shear [17].

**Figure 1:**
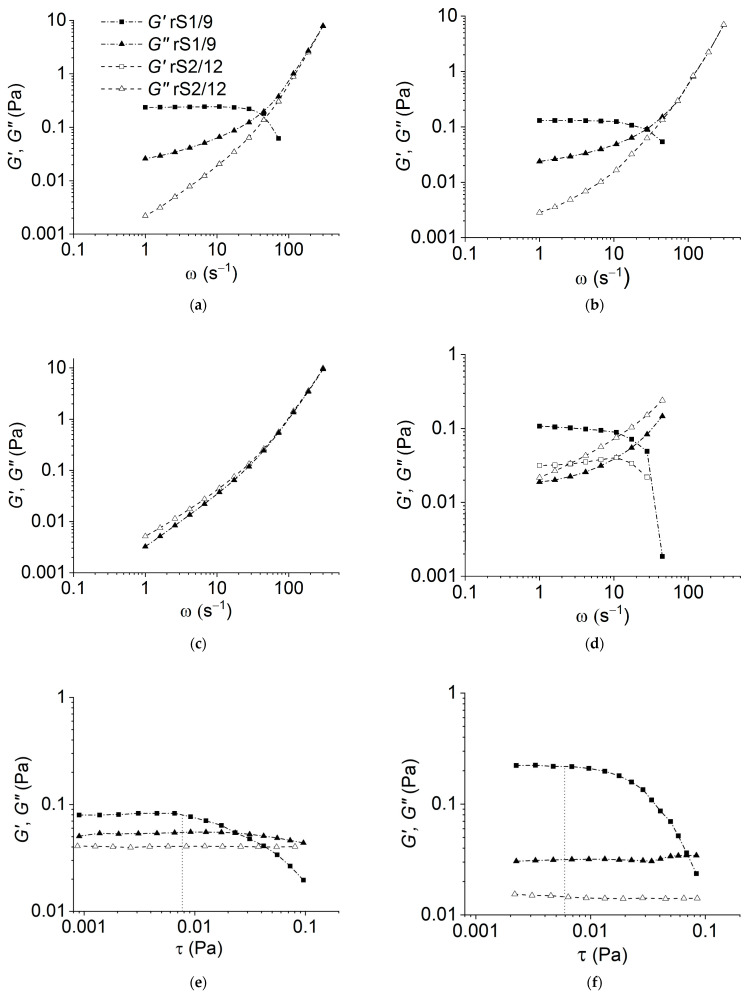
Dependence of the storage (*GG*′) and loss (*G*″) moduli on frequency for the dispersions rS1/9 and rS2/12 with a concentration of 3 mg/mL in (**a**) 2% and (**b**) 5% formic acid solutions; (**c**) in HFIP; and (**d**) with a concentration of 11 mg/mL in 5% formic acid solutions. Estimation of the yield stress value for the dispersions rS1/9 with a concentration of 3 mg/mL in (**e**) 2% and (**f**) 5% formic acid solutions. The value of the yield strength is indicated with a vertical dotted line.

As can be seen in Figure 1a,b, the rS1/9 dispersions at a low concentration (3 mg/mL) in 2% and 5% formic acid solutions behave like weakly structured liquids. However, the rheological behavior of the rS2/12 dispersions with the same concentration in 2% formic acid solution corresponds to a viscous liquid model (*G*′<<*G*″) in the entire frequency range (Figure 1a,b). An increase in the protein concentration leads to the structuring of the dispersions (Figure 1d). It should be noted that the value of the storage modulus *G*′ for rS1/9 is greater than that for rS2/12 in the entire frequency range (Figure 1d). Thus, one can conclude that the structuring process in rS1/9 dispersions proceeds more intensively than in rS2/12 dispersions. The strength of forming gel can be estimated using the yield stress value (in Figure 1e,f, the estimated value is indicated with a vertical line). As can be seen, the resulting gels have a low strength of less than about 0.01 Pa, which weakly depends on the content of formic acid in the studied concentration range. However, in HFIP, the dispersions of both proteins demonstrate the properties of a viscous liquid (Figure 1c).

**Figure 2:**
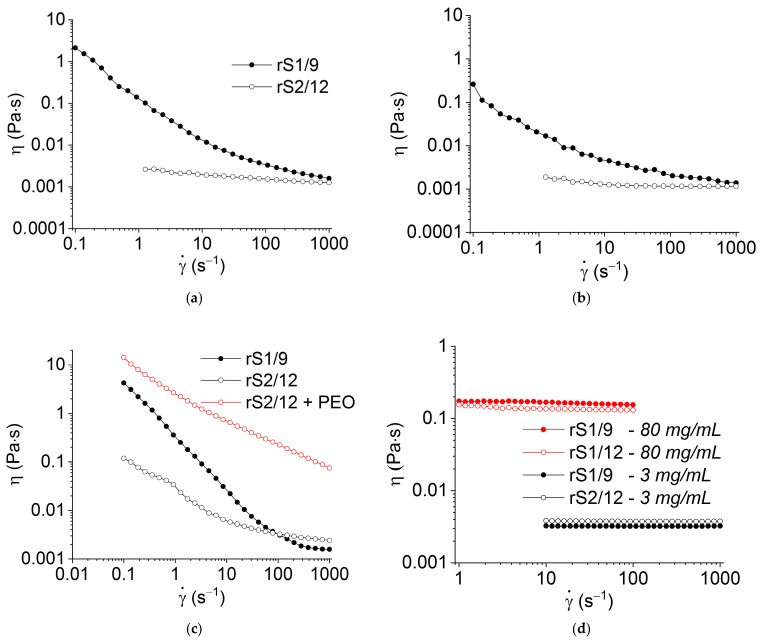
Flow curves of the rS2/12 and rS1/9 dispersions with a concentration of 3 mg/mL in (**a**) 2% and (**b**) 5% formic acid solutions; (**c**) 11 mg/mL in 5% formic acid solution with and without PEO; and (**d**) in HFIP with concentrations of 3 mg/mL and 80 mg/mL.

The results of the RS dispersion rheological measurements in shear flow are shown in Figure 2. In contrast to the rS2/12 dispersions, the rS1/9 dispersions with a concentration of 3 mg/mL in 2% and 5% formic acid solutions are structured, as evidenced by the non-Newtonian flow behavior (Figure 2a,b). However, as the concentration increases to 11 mg/mL, the rS2/12 dispersion in a 5% formic acid solution also begins to acquire a non-Newtonian flow character (Figure 2c). It should be noted that at high shear rates, as a result of the applied shear stress, the structure of both dispersions is destroyed, and the system begins to flow like a Newtonian fluid (Figure 2c).

To improve the fiber-forming properties of the rS2/12 aqueous dispersion with a concentration of 11 mg/mL, PEO (19 mg/mL) was added. As can be seen in Figure 2c, the addition of PEO led to a significant increase in viscosity, which should ensure a stable electrospinning process.

As in the regime of periodic oscillations, the HFIP solutions of both RS with a concentration of 3 mg/mL behave like Newtonian liquids (Figure 2d), and even an increase in concentration to 80 mg/mL did not lead to the formation of a three-dimensional network structure (Figure 2d).

**Figure 3:**
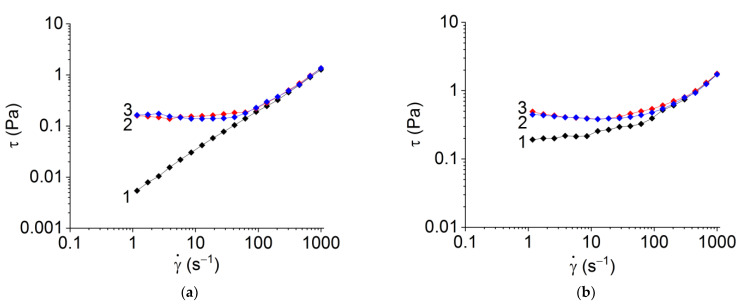
Appearance of yield stress for the dispersions of (**a**) rS2/12 and (**b**) rS1/9 with a concentration of 3 mg/mL in a 5% formic acid solution during successive shear cycles (from 1 to 3). The number of cycles is indicated by each line.

It should be noted that the rheological properties of RS dispersions in formic acid solutions significantly depend on the number of shear cycles and shear rate, e.g., at the second and third cycles, a pronounced thixotropic effect with a yield strength of about 0.1–0.5 Pa is observed (Figure 3).

Despite the fact that the exact value of yield stress depends on the method of its determination (cf. Figure 1e,f and Figure 3), the effect of structure destruction is evident.

One can suggest that the observed behavioral evolution of the RS aqueous dispersions is associated with the formation and interaction of the protein nanofibrils due to applied shear stress and the development of a rare network of nanofibril entanglements.

### 3.2. Self-Assembly of RS

**Figure 4:**
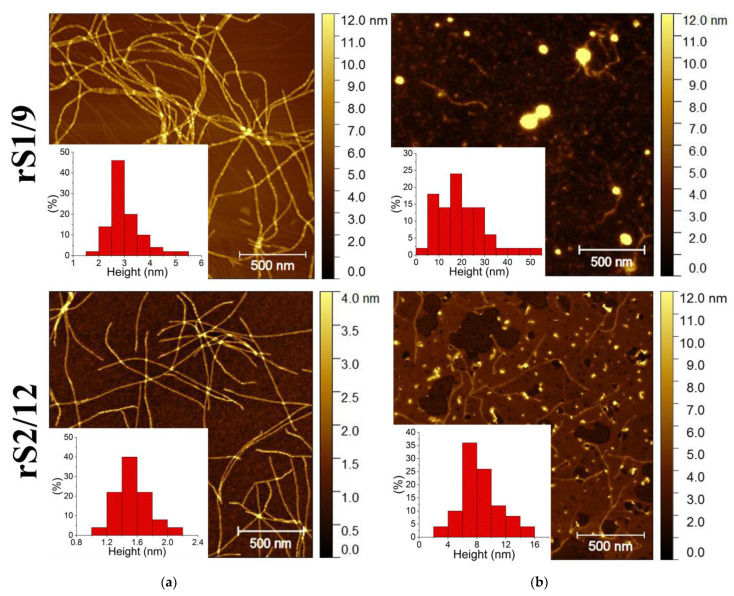
AFM images of the rS1/9 (the first row) and rS2/12 (the second row) nanofibrils and spherical aggregates prepared from the dispersions with a concentration of 0.08 mg/mL in (**a**) formic acid solutions and (**b**) HFIP.

Atomic force microscopy (AFM) reveals the solvent effect on the aggregation of the protein molecules in the dilute rS1/9 and rS2/12 dispersions (Figure 4).

In aqueous solutions of formic acid, regardless of the type of RS and its concentration, only fibrillar structures were observed. The rS1/9 nanofibrils were very long (up to a few micrometers), whereas the rS2/12 nanofibrils were notably shorter (Figure 4a). Moreover, the rS2/12 fibril height was approximately two times lower than that for rS1/9.

The morphology of the RS aggregates in HFIP is quite different. Spidroin rS1/9 deposited on the mica substrate from HFIP forms round particles with a wide range of lateral diameters (from 20 to 100 nm) and heights (from 7 to 50 nm). In addition, a few individual fibrils of two different sizes can be observed, i.e., long and high (about 3.5 nm in height and up to several micrometers in length) fibrils and very short (about 2 nm in height and up to 100 nm in length) ones. The rheological tests of the protein dispersions did not change the observed morphology. The morphology of the rS2/12 dispersions was quite similar. The globular structures with a diameter in the range from 10 to 60 nm and a height from 3 to 16 nm, with the most probable height of about 4.5 nm, were observed.

The size of the nanofibrils is consistent with the results of other studies. For example, the height of nanofibrils of the recombinant spidroin ADF4(C16) in aqueous solutions was reported to be in the range from 2.5 to 10 nm, whereas their length was in the range from 200 nm to 1 µm [18,19].

**Figure 5:**
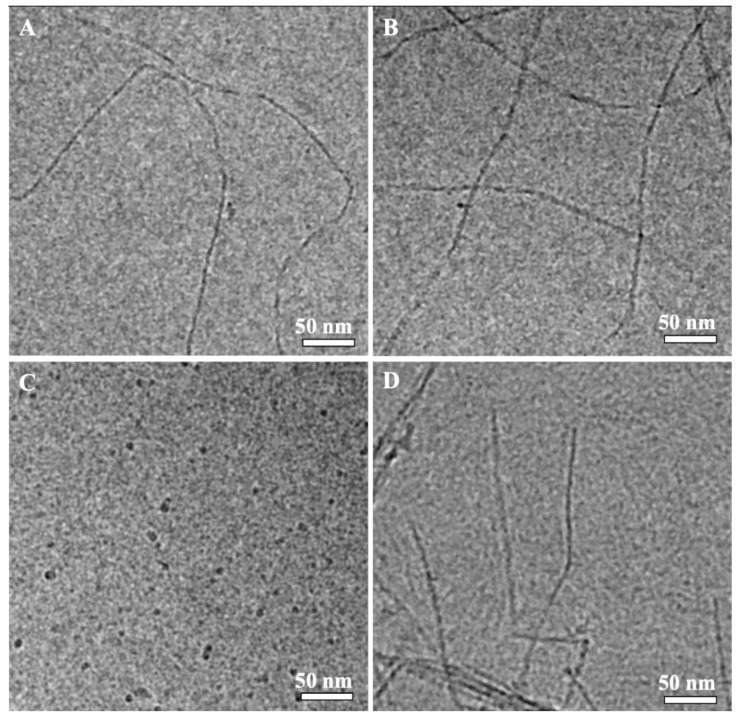
Cryo-TEM images of the nanofibrils and spherical aggregates in the (**A**,**B**) rS1/9 and (**C**,**D**) rS2/12 dispersions with a concentration of 3 mg/mL in a 5% formic acid solution (**A**,**C**) before rheological tests; (**B**,**D**) after rheological tests.

Cryo-TEM images of the rS1/9 and rS2/12 dispersions with a concentration of 3 mg/mL in a 5% formic acid solution before and after rheological tests are shown in Figure 5. As can be seen, the particles of the rS1/9 aqueous dispersions retain fibrillar morphology after shear during rheological measurements. The rS2/12 particles formed in formic acid solution are spherical, with diameters in the range of 3 to 10 nm. Nanofibrils in the cryo-TEM images of the rS2/12 aqueous dispersions were found only after rheological treatment. The average fibril diameter is about 5 nm.

**Figure 6:**
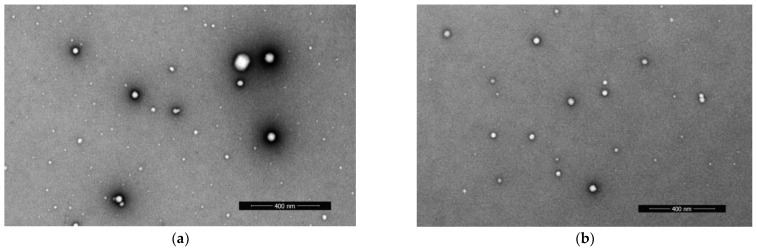
SEM images of spherical particles in the (**a**) rS1/9 and (**b**) rS2/12 dispersions in HFIP with a concentration of 1 mg/mL.

The rS1/9 and rS2/12 proteins in HFIP assemble into spherical particles. The diameter of the rS1/9 spherical particles was evaluated in the range from 6 to 128 nm, whereas the rS2/12 particles have diameters in the range from 7 to 61 nm (Figure 6).

AFM, SEM, and cryo-TEM reveal fibrillar morphology of the RS aggregates in formic acid aqueous solution and only spherical morphology in HFIP. Thus, one can suggest that the protein fibrils form a three-dimensional network of entanglements in formic acid aqueous solution, resulting in non-Newtonian behavior of these dispersions, whereas the RS dispersions in HFIP behave like a Newtonian liquid, which can be associated with the spherical morphology of the RS aggregates.

### 3.3. Electrospinning of the RS Nanofibers

**Figure 7:**
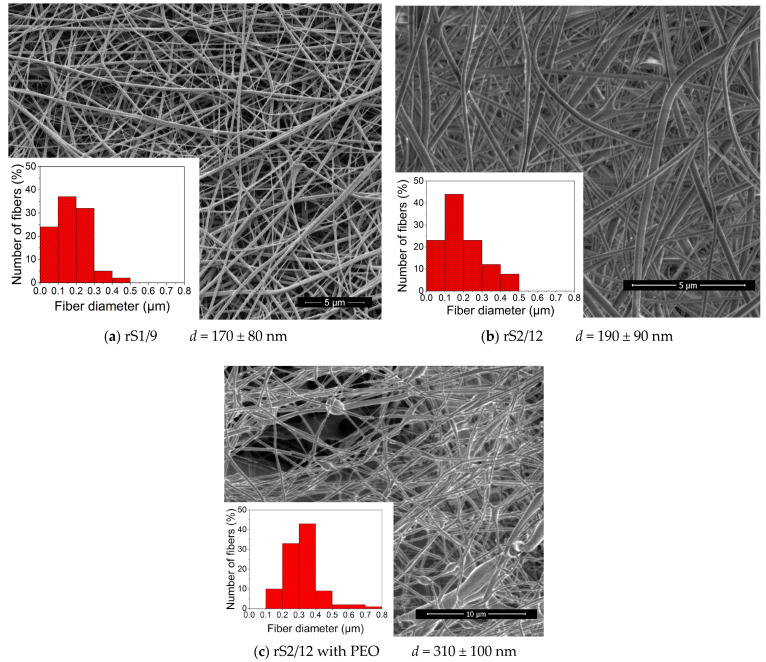
SEM images of the fibrous materials formed from the RS dispersions (**a**,**b**) in HFIP and (**c**) in formic acid solution. The corresponding histograms of fiber diameter distribution are shown in the inserts.

Based on the previous studies [20] and the results of rheological tests, the fiber-forming concentration of RS in formic acid solution (11 mg/mL for rS2/12) and in HFIP (80 mg/mL) was selected. The SEM images of nonwoven materials formed from the dispersions in HFIP are shown in Figure 7a,b. One can note the wide diameter distribution of the fibers, which can be explained by the splitting of the primary jet into sub-jets [21].

Due to the limited solubility of rS1/9 and rS2/12 in water, a fiber-forming additive, i.e., PEO, was used. The minimum concentration of PEO in an aqueous medium (19 mg/mL) was chosen based on the literature data in ref. [22], where it was demonstrated that such a concentration enables the formation of defect-free fibers. The addition of PEO to an aqueous rS2/12 dispersion led to a significant increase in viscosity (Figure 2c), resulting in an increase in the fiber diameters. One can observe some defects in the SEM images of the fibers, such as “gluing” and “flattening”, indicating only partial compatibility of the components in the spinning solution (Figure 7c). The addition of PEO to the aqueous rS1/9 dispersion resulted in gel formation, preventing the electrospinning process. Thus, the possibility of fibrous RS material formation by electrospinning from various solvents was demonstrated. However, for various biomedical applications, it is necessary to evaluate the conformational state of the proteins in fabricated materials.

### 3.4. IR Spectroscopy of the Films and Nonwovens

Proteins are organized commonly in complex three-dimensional structures consisting of local regions with different spatial configurations, e.g., β-turns, β-sheets, non-oriented structures, and α-helices. In these conformations, the C=O bond has different vibration frequencies, and, as a result, the resulting IR spectrum consists of several overlapping components since the width of these absorption bands is greater than the distance between the maxima of neighboring peaks. Therefore, to obtain insight into protein structure based on the IR spectroscopy data, it is necessary to carry out mathematical data processing of the experimental spectra. To analyze the conformational state of the RS proteins, the deconvolution procedure of the absorption band in the Amide I region was used.

**Figure 8:**
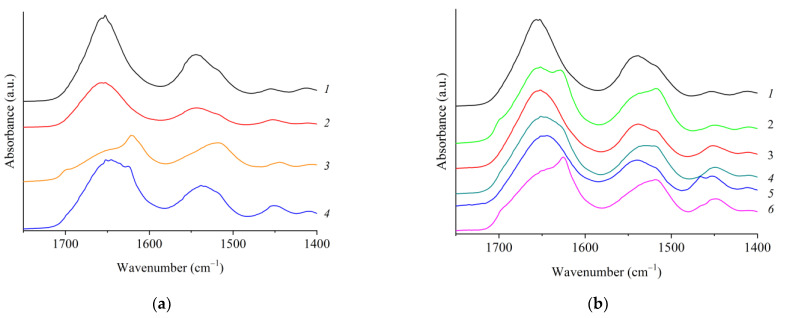
(**a**) IR spectra of the rS1/9 (odd numbers) and rS2/12 (even numbers) films cast from their dispersions in HFIP (1, 2) and in formic acid solutions (3, 4); (**b**) IR spectra of the rS1/9 electrospun materials from the dispersion in HFIP (1, 2). IR spectra of the rS2/12 electrospun materials from the dispersions in HFIP (3, 4) and in a 2% formic acid solution (5, 6). Odd numbers indicate samples without ethanol treatment. Even numbers correspond to the samples after their treatment with ethanol.

The IR spectra of the rS1/9 and rS2/12 films and fibrous materials are shown In Figure 8.

The films and nonwoven materials are characterized by the presence of absorption bands typical of nitrogen-containing compounds [23,24,25,26], i.e., 3276 cm^−1^ (stretching vibrations of N–H bonds), 1639 cm^−1^ (Amide I band—stretching vibration C=O), 1526 cm^−1^ and its overtone at 3072 cm^−1^ (Amide II band—bending vibrations N–H and stretching C–N), 1205–1280 cm^−1^ (Amide III band—N–H bending vibrations + C=O bending vibrations + C–C stretching vibrations). However, significant differences can be observed between the spectra of the films cast from the RS dispersions in formic acid solutions and in HFIP, as well as between the nonwoven materials in the region of the Amide I absorption band (1600–1700 cm^−1^).

**Figure 9:**
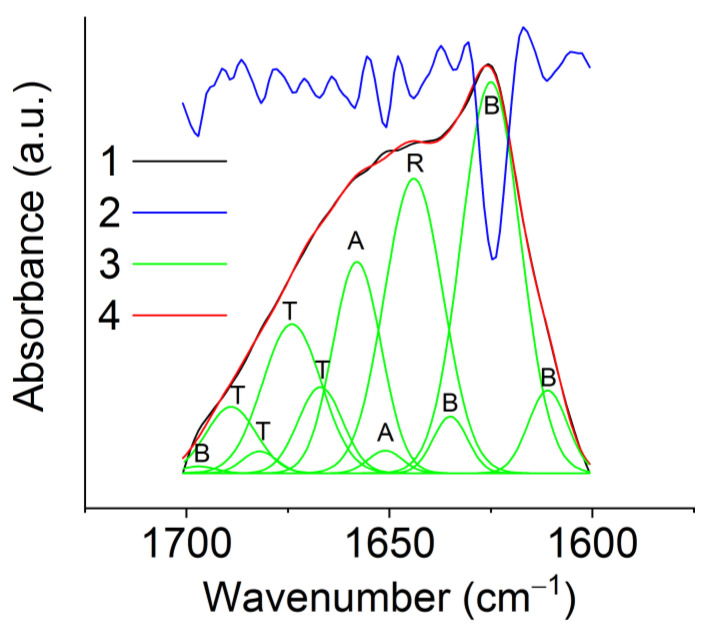
Deconvolution analysis of the Amide I absorption band in the infrared spectrum of the rS2/12 nonwoven material formed from the 5% formic acid solution after ethanol treatment. (**1**) Experimental IR spectrum; (**2**) its second derivative; (**3**) individual deconvoluted peaks marked as α-helices (A), β-sheets (B), random coils (R), and β-turns (T); (**4**) the summation of the individual peaks.

Spidroin molecules have various conformations, e.g., β-turns (1661–1691 cm^−1^), β-sheets (1609–1641 cm^−1^), and antiparallel β-sheets (1695 ± 4 cm^−1^), unoriented structures (random coils) (1644–1648 cm^−1^) and α-helices (1650–1660 cm^−1^) [24,25,26,27,28]. These conformations can be identified in the IR spectra in the Amide I absorption band region. As an example, the deconvolution analysis for the rS2/12 nonwowen is shown in Figure 9. The results of this analysis for rS1/9 and rS2/12 films are given in Table 1. Comparing different samples, one can conclude that the fraction of β-sheets dramatically increases in the aqueous solutions compared to that in HFIP (almost 1.5 times for rS2/12 and 2.2 times for rS1/9), whereas the total fraction of random coils, α-helices, and β-turns decreases. It should be noted that this effect is more prominent for rS1/9 compared to rS2/12.

The deconvolution analysis of IR spectra reveals similar conformational compositions of the nonwoven materials and films produced from the dispersions in HFIP for both proteins (Table 1 and Table 2). 

As is known, the treatment of RS-based materials with ethanol promotes the formation of β-sheets in these proteins (Figure 8b) [29]. This effect for nonwoven materials is shown in Table 2. It is noteworthy that the conformational state of both proteins becomes nearly the same after treatment with ethanol.

Thus, it was shown that the solvent affects the conformational composition of the recombinant spidroins that can be used for the formation of the nanofibers with the desired properties.

## 4. Discussion

The effect of solvent, amino acid composition, and protein concentration on the rheological properties of the RS dispersions was revealed. The rheological behavior of the RS rS1/9 and rS2/12 dispersions is similar. However, the formation of fibrillar structures is facilitated in the rS1/9 dispersions. The width and height of nanofibrils formed from this spidroin are approximately 2 times greater compared to rS2/12. The formation of fibrillar morphology is promoted in aqueous solutions of formic acid, whereas spherical structures are formed in HFIP. Thus, a three-dimensional network of entanglements appears in the aqueous RS dispersions, resulting in their non-Newtonian flow behavior. When the critical gelation concentration of 50 mg/mL was reached, the aqueous dispersions of both proteins lost their ability to flow. It is noteworthy that the natural spinning solution isolated from the large ampulla glands of the spider *Nephila senegaliensis* [30] is also a non-Newtonian liquid. In addition, a similar result was reported in ref. [31], where a solution of regenerated spider silk turned into a gel with a decrease in pH from 8 to 4.8 and an increase in concentration from 20 to 35%. This transition was associated with an increase in the fraction of β-sheets in the conformational composition of spidroin. Thus, a comparative analysis of the rheological behavior of RS dispersions under shear deformations and in the oscillatory mode reveals the effect of solvent on the gel transition as the protein concentration increases. The formation and destruction of a continuous three-dimensional fibrillar network structure in the dispersions under shear stress were shown.

Recently, it has been realized that the protein folding process cannot be described simply as a consequence of individual protein molecule translations, depending only on intramolecular primary, secondary, and tertiary interactions. Instead, it is assumed that the entire population of protein molecules is a conglomerate of several classes of assemblies that undergo folding in similar or different ways. As a result, the same protein molecules can exist in different folded conformers [32].

Although the formation of differentially folded conformations depends on the free energy and entropy changes of the molecules, their lifetime in the folded intermediate form is determined by kinetic parameters. The transition from one folded conformer to another is due to energy jumps. Energy, usually in the form of heat, comes from the protein’s environment. Apparently, with increasing temperature and pressure, water molecules destroy the interaction of hydrophobic regions in protein molecules, which leads to their dissolution. Thus, the use of a microwave oven to set high temperatures and pressures made it possible to achieve enormous values of the solubility of rMaSp1 and rMaSp2 RS in an aqueous medium [13].

The chemical nature of the solvent also affects the aggregation process and the conformational state of spidroins. The solubility of proteins in water is due to solvation of the COO^−^ and NH_3_^+^ groups, which carry an ionic charge in alkaline or acidic conditions.

HFIP is a strong polar solvent capable of dissolving fibrillar proteins such as collagen, elastin, fibroin, keratin, and spidroin [33,34,35,36]. On the one hand, the protein molecule tends to adopt the most favorable conformation with the minimum free energy, and, on the other hand, the solvent forces it to adopt the conformation with the lowest free energy. Thus, the secondary and tertiary structures of proteins in solution will be stabilized due to the competition of these two factors [37]. For this reason, in solutions of dilute alcohols, proteins can take the form of both α-helix and β-sheets, while with an increase in the hydrocarbon radical and the degree of alcohol halogenation, the probability of adopting the α-helix conformation increases.

Our results are in agreement with the literature. The fraction of β-sheets in an aqueous medium is noticeably higher than that in HFIP, but the total content of the random coils, α-helices, and β-turns decreases, which causes the formation of fibrils. A similar effect of the solvent was reported in a number of publications devoted to the physical and chemical properties of RS. For example, in ref. [38], the fibers of aciniform spidroin produced by wet spinning from 20% formic acid contained 40% β-sheets. When the fibers were formed from a Tris-buffered saline solution (pH = 7.5), their crystallinity reached 60% [39]. Some types of fibers of *N. edulis* and *A. avicularia* spiders also have a high (>60%) content of β-sheets, which makes them promising for the formation of nervous tissue using tissue engineering methods [40]. The fraction of β-sheets in the materials formed from HFIP is usually lower, i.e., 10–15% [41,42], but it can be increased up to 30–40% using their treatment with alcohols such as methanol, ethanol, and isopropanol. For the rS1/9 spidroin, the observed changes are more distinct compared to rS2/12 due to a significant difference in the amino-acid sequences of these proteins, namely in the length of the poly-Ala blocks capable of forming β-sheets. 

Comparison of the IR spectra of films and fibrous materials showed the absence of significant conformational changes in spidroins caused by jet stretching during electrospinning from both HFIP and formic acid solutions (Table 1 and Table 2).

It was reported that the mechanical properties of protein fibers can be controlled by varying the number and length of poly-Ala blocks responsible for crystallinity. Theoretical calculations show [43] that an increase in the length and a decrease in the number of poly-Ala blocks lead to an increase in the strength and elastic modulus of fibers. In our study, the length of poly-Ala blocks in rS1/9 is greater than that in rS2/12 (7.5 and 6.2 Ala residues, respectively), and their number is smaller (36 and 60 per molecule, respectively), which gives the possibility to assume the presence of higher mechanical properties of materials based on rS1/9.

## 5. Conclusions

The comparative analysis of the rheological behavior of the rS1/9 and rS2/12 dispersions under shear deformations and in the oscillatory mode reveals the influence of the solvent on the gel transition with increasing protein concentration. The formation of a continuous, three-dimensional fibrillar network structure under shear stress was demonstrated.

The nanofibrous materials from the rS1/9 and rS2/12 dispersions in HFIP were fabricated by electrospinning. It should be noted that for the preparation of nonwoven materials with high mechanical properties, it is desirable to form them from formic acid solutions [44]. However, due to the high structure-forming ability of RS in water, their solutions retain the ability to flow only at low concentrations. Therefore, in order to produce the fibers from aqueous solutions of RS, the process of their electrospinning should be carried out at higher protein concentrations in the spinning solutions. Moreover, the spinning conditions and the solvent ensuring the fluidity of the spinning solution should be carefully selected, for example, as described in ref. [45] devoted to the production of collagen fibers.

## Figures and Tables

**Table 1:** Conformational composition of the RS rS1/9 and rS2/12 films cast from HFIP and formic acid solutions.

Spidroin	Conformation	From HFIP	From Formic Acid Solution
**rS1/9**	β-turns	29	12
	β-sheets	31	69
	random coils	11	2
	α-helices	29	17
**rS2/12**	β-turns	30	24
	β-sheets	27	40
	random coils	14	10
	α-helices	29	26

**Table 2:** Conformational composition of the RS rS1/9 and rS2/12 nonwoven materials formed from the dispersions in HFIP or in formic acid solution before and after their treatment with ethanol (4 h at 60 °C).

Spidroin	Conformation	From HFIP	From HFIP, after Ethanol Treatment	From Formic Acid Solution	From Formic Acid Solution; after Ethanol Treatment
**rS1/9**	β-turns	33	23	-	-
	β-sheets	31	39	-	-
	random coils	9	31	-	-
	α-helices	27	7	-	-
**rS2/12**	β-turns	29	23	30	24
	β-sheets	30	40	37	43
	random coils	19	24	12	23
	α-helices	22	13	21	10

## Data Availability

The data presented in this study are available upon request from the corresponding author.
